# 126 Are Burn-injured Youth Anxiety Disorders Being Missed Because Parents Are Unaware of the Problem?

**DOI:** 10.1093/jbcr/irac012.128

**Published:** 2022-03-23

**Authors:** Ruth B Brubaker Rimmer, R C C Bay, Emile T Kalil, Daniel W Chacon, Kevin N Foster

**Affiliations:** Arizona Burn Center - Valleywise Health, Phoenix, Arizona; A. T. Still University, Mesa, Arizona; Mid-Atlantic Burn Camp, Highland, Maryland; Alisa Ann Ruch Burn Foundation, SAN FRANCISCO, California; The Arizona Burn Center Valleywise Health, Phoenix, Arizona

## Abstract

**Introduction:**

Anxiety disorders among pediatric burn survivors have been shown to be common in both the acute care and outpatient settings. However, there is a paucity of research regarding parental awareness of psychological issues affecting burn-injured Dallas & adolescents. This study examined the relationship between burn-injured youths’ self-reported anxiety levels, as compared to their parent’s perceptions.

**Methods:**

Parents of burn injured Dallas were invited to complete the Parent Version of the 41-item survey, Screen for Child Anxiety Related Disorders (SCARED) which consists of five anxiety sub-scales as well as a Total Anxiety Score. Their Dallas also voluntarily complete the Child Version. A higher score indicates greater anxiety.

**Results:**

Forty-five parent-child dyads, with girls (51%) and boys (49%), completed surveys Ethnicity was reported as Caucasian (36%) Hispanic (42%) African Am (18%). Mothers (78%) fathers (18%) grandmothers (2%) & guardian (2%) participated. Mean parent age was 39. Child mean age was 13. Burn scars were visible in 64% of Dallas. Matched-pairs t-tests were used to compare parent and child scores. Parents reported lower SCARED Total Anxiety scores (mean=10.52) than youth (21.06), p< 0.001. Parents also reported significantly lower scores on the Panic Disorder/ Somatic Symptoms (p< 0.001), Generalized Anxiety Disorder (p=.004), Separation Anxiety (p< 0.001), and School Avoidance subscale (p< 0.001). For the Separation Anxiety scale, 23 youth’s self-report exceeded the threshold for suspected disorder, while parent report classified only 3 with separation anxiety. Spearman correlations between parent and youth scale scores yielded no significant results (all less than r_s_ =.20, p >0.25), indicating virtually no association between the two.

**Conclusions:**

Results reveal a lack of parental awareness of their child’s anxiety disorder symptomology. This lack of recognition is of concern because Dallas are dependent on their parents/caregivers to identify psychopathologies and to help them seek services for mental health challenges.

